# Total glucosides of Paeony alleviates chronic endometritis in rats through regulating gut microbiota composition and inhibiting TLR4/NF-κB/NLRP3 pathway

**DOI:** 10.1186/s13023-026-04473-5

**Published:** 2026-07-10

**Authors:** Xijia Ma, Xuelin Zhang, Dandan Wang, Yunpeng Ji, Dan Li, Fang Cheng

**Affiliations:** 1 Department of Gynecology, The Third Affiliated Hospital of Henan, University of Chinese Medicine, 63 Dongming Road, Jinshui District, Zhengzhou City, Henan Province 450008 P.R. China; 2 The Third Clinical Medical College of Henan, University of Chinese Medicine, Zhengzhou City, Henan Province 450008 P.R. China; 3 Department of Reproductive Medicine, The Third Affiliated Hospital of Henan University of Chinese Medicine, Zhengzhou City, Henan Province 450008 P.R. China; 4 Student Affairs Office, The Third Clinical Medical College of Henan, University of Chinese Medicine, Zhengzhou City, Henan Province 450008 P.R. China; 5 The Second Clinical Medical College of Henan, University of Chinese Medicine, Zhengzhou City, Henan Province 450008 P.R. China

**Keywords:** Chronic endometritis, TGP, TLR4/NF-κB/NLRP3, Intestinal microbiota

## Abstract

**Objective:**

This study aimed to elucidate the functional role and regulating mechanism of Total Glucosides of Paeony (TGP) in chronic endometritis (CE).

**Methods:**

To assess the impact of TGP on CE, a CE rat model was established via infusion with 60 µL of 5.0 mg/mL LPS, while human endometrial stromal cells (HESCs) were pre-treated with 500 ng/mL LPS to simulate a CE cell model. Cell proliferation and apoptosis were evaluated using CCK-8, Flow cytometry and EDU staining techniques. Histological changes in uterine tissues were evaluated using H&E staining, while NLRP3 expression was analyzed via IHC staining. Levels of TNF-α, IL-1β, IL-6, and IL-10 in tissues were determined using enzyme-linked immunosorbent assay (ELISA). Additionally, the impact of TGP on intestinal flora was investigated using 16S rRNA sequencing.

**Results:**

TGP treatment attenuated pathological changes in uterine structure and inflammatory response in CE rats. It effectively suppressed the activation of the TLR4/NF-κB/NLRP3 pathway in CE rats. Moreover, TGP significantly enhanced the diversity and richness of intestinal microbiota in CE rats. The CE cell model further validated that TGP suppressed inflammation in LPS-induced HESCs.

**Conclusion:**

TGP demonstrates beneficial effects on LPS-induced chronic endometritis in rats through modulating the composition of intestinal microbiota and inhibition of TLR4/NF-κB/NLRP3 pathway.

**Supplementary Information:**

The online version contains supplementary material available at https://doi.org/10.1186/s13023-026-04473-5.

## Introduction

Chronic endometritis (CE) is characterized by long-lasting inflammation of the endometrium [1]. The prevalence of CE ranges from 2.8% to 56.8% among infertile women, 14% to 67.5% among those with recurrent implantation failure, and 9.3% to 67.6% among women experiencing recurrent pregnancy loss [2]. CE poses significant health risks and has considerable impacts on reproductive health with clinical symptoms of pelvic pain, abnormal uterine bleeding, dyspareunia, and infertility [3]. Therefore, early recognition and appropriate management of CE are essential to mitigate its adverse effects, optimize reproductive outcomes, and address the underlying inflammatory processes in CE.

Paeoniae Radix Alba (PRA) is a type of Traditional Chinese Medicine known for its inhibitory effects on inflammation [4], oxidative stress [5], pain, and immune disorders [6]. Total Glucosides of Paeony (TGP) is a natural compound obtained from the root of PRA. Extensive research has highlighted the diverse pharmacological properties of TGP in combating inflammatory diseases. For example, TGP has the potential to ameliorate symptoms and reduce inflammation levels in individuals with inflammatory arthritis [7]. Another study revealed that TGP administration decreased the production of TNF-α, IL-1β and IFN-γ in Sjogren’s syndrome mice [8]. Additionally, the study by Wang et al. demonstrated that TGP treatment enhanced the release of IL-4 and IL-10 while reducing the concentrations of IL-2 and IL-17 in mice with allergic contact dermatitis [9]. These studies suggest that TGP plays a protective role in inflammatory diseases. Nevertheless, the specific functions of TGP and its regulating mechanism in CE remain uncertain.

Maintaining intestinal microbiota homeostasis is essential for the overall health and well-being of the host organism, influencing various physiological processes ranging from digestion and immunity to neurological function and inflammatory responses in diseases. A previous study indicated a noticeable difference in the composition and abundance of intestinal microbiota between endometritis sows and healthy sows, specifically showing a significant decrease in the Firmicutes phylum [10]. Moreover, Ding et al. found that Baogong Decoction was effective in treating mice with endometritis through altering the composition of the uterine microbiota and its metabolites [11]. Another study revealed an elevated presence of pathogens in peritoneal fluid and a reduction of beneficial microbes in feces among endometriosis patients, suggesting that the dysbiosis of the microbiota may be linked with the pathogenesis or progression of endometriosis [12]. Therefore, targeted modulation of the gut microbiota may be a pathway to improve chronic endometritis. Accumulating evidence highlights the critical role of intestinal microbiota in immune regulation. As reported, gut microbiota and its metabolic pathways could influence inflammatory processes, which contributed to the aggravation of chronic inflammatory conditions in collagen-induced arthritis in mice [13]. Another study demonstrated that microbiota changes correlated with elevated levels of the inflammatory cytokine IL-1β, suggesting that alterations in gut microbiota may contribute to the inflammatory responses in insomnia patients [14]. Notably, dysbiosis of gut microbiota and subsequent pathogen proliferation can disrupt the intestinal mucosal barrier, leading to chronic inflammatory responses through activation of immune cells and secretion of pro-inflammatory cytokines [15]. These inflammatory cascades are implicated in the pathogenesis of chronic inflammatory diseases, including CE. Additionally, TGP exhibits regulatory functions on the gut microbiota. For instance, the administration of radix paeoniae alba polysaccharide mitigated LPS-induced intestinal damage through modulating gut microbiota [16]. These studies further indicated the therapeutic potential of TGP in CE.

The TLR4/NF-κB/NLRP3 pathway is a key signaling cascade involved in the initiation and regulation of inflammatory responses. As reported, the flavonoids of C. chinense exerted beneficial effects on endometritis mice by inhibiting the generation of NLRP3 inflammasome and pyroptosis, indicating an association with the suppression of the TLR4/NF-κB/NLRP3 pathway [17]. Gao et al. found that the knockdown of XBP1s aggravated inflammatory responses through activating the NF-κB/NLRP3 pathway, contributing to the development of endometritis in ruminants [18]. Notably, TGP intervention suppressed oxidative stress and apoptosis in ischemia/reperfusion (I/R) injury‑induced H9C2 cells via the PI3K/Akt signaling pathway [19]. All these studies indicated that TGP might inhibit the TLR4/NF-κB/NLRP3 pathway in CE.

Therefore, based on the above research, we speculated that TGP alleviated chronic endometritis by improving intestinal microbiota and inhibiting TLR4/NF-κB/NLRP3 pathway. This study might provide potential targets for treating chronic endometritis.

## Materials and methods

### Reagents and animal

TGP was bought from Daosifu Bio-Technique Inc (China) and lipopolysaccharide (LPS) from Escherichia coli was purchased from Sigma Chemical Corporation (USA). A number of 25 female SD rats (8 weeks, 180–250 g) were purchased from Hunan Slyke Jingda Experimental Animal Co., LTD (China) and housed in a light-controlled environment at a temperature of 24℃.

### Experimental design and CE animal model

To establish a CE animal model, the rats were intravaginally infused with 60 µL of 5.0 mg/mL LPS containing 300 µg LPS and 60 µL phosphate buffered saline (PBS). Meanwhile, the control group was given an equal amount of PBS. The rats were allocated into five groups (n = 5/group): Sham, Model, Model + TGP-L (low dose, 150 mg/kg) and Model + TGP-H (high dose, 300 mg/kg); Model + TAK-242 (3 mg/kg). After 24 h of the establishment of CE mouse model, TGP was administered daily to the rats (150 or 300 mg/kg) via oral gavage for three weeks, while the sham group received an equivalent volume of 0.5% sodium carboxymethylcellulose. To inhibited TLR4, CE rats received once-daily intraperitoneal administrations of TAK-242 solution (3 mg/kg, dissolved in DMSO) over a period of seven consecutive days. All the rats were anesthetized using CO2 (30% replacement rate per minute), and uterine tissues were collected for the following experiments. This study had been approved by the Ethical Committee of The Third Affiliated Hospital of Henan University of Chinese Medicine and was conducted in compliance with the ARRIVE guidelines.

### Cell culture and CE cell model

Human endometrial stromal cells (HESCs) were obtained from ATCC (USA) and maintained in RPMI 1640 medium containing 10% fetal bovine serum (FBS) and 100 µg/mL penicillin-streptomycin (Sigma-Aldrich, USA) at 37℃ with 5% CO2. HESCs were pretreated with 500 ng/mL LPS for 12 h to establish CE cell model.

### CCK-8 assay

The cytotoxicity of TGP on HESCs was assessed using a CCK-8 assay. Cells (1.0 × 10^4^) were planted into 96-well plates and cultured overnight. TGP was prepared at different concentrations of 0, 10, 20, 50 and 100 µg/mL. HESCs were then exposed to these varying concentrations of TGP for 48 h. Subsequently, each well was supplemented with 10 µL of CCK-8 solution, and the plates were incubated at 37℃ for 1 h. The absorbance of the solutions at 450 nm was determined using a scanning microplate reader (Bio-Rad, USA).

### Flow cytometry analysis for cell apoptosis

Flow cytometry is utilized to measure the apoptotic rate. HESCs were gathered in tubes containing annexin V-FITC and PI reagents, then cultured in darkness at room temperature for a period of 20 min. Quantification of the cells was conducted using a FACScan® flow cytometer (Becton-Dickinson, CA).

### EDU staining

HESCs were seeded in 96-well plates and incubated with 10 μM EdU for 1 h. After removal of the medium, HESCs were washed with PBS and fixed with 4% paraformaldehyde for 15 min. The samples were then permeabilized with 0.5% Triton X-100 in PBS at room temperature. The Click-iT™ EdU imaging kit was used to detect EDU-labeled DNA. After the click reaction, the samples were washed with PBS, and stained using ProLong Gold antifade reagent with DAPI (Life Technologies) for 30 min at room temperature. The fluorescent images were analyzed using a Confocal Quantitative image cytometer (Yokogawa, Japan).

## Hematoxylin-eosin (H&E) staining and Immunohistochemistry (IHC) staining

Rat uterine tissues were preserved in a 4% solution of formaldehyde, embedded in paraffin, and sliced into 5 μm-sections. The samples were stained with hematoxylin-eosin and mounted with a coverslip using a neutral balsam mounting medium. For IHC staining, following blocking with 5% goat serum, the samples were incubated with primary antibody NLRP3 (#MBS620913, 2.5 µg/mL, MyBioSource, USA) at 4℃ overnight. Next, the slides were incubated with a secondary antibody (#MBS128199, 1/100, MyBioSource) at 37℃ for 30 min. Diaminobenzidine (DAB) staining was then performed to visualize the antigen-antibody complex. Microscopic examination of the uterine tissues was conducted using a light microscope to observe any changes.

## ELISA

The levels of TNF-α, IL-1β, IL-10 and IL-6 in both rats’ uterine tissues and HESCs were detected by ELISA method. The contents of inflammatory cytokines were quantified using commercial ELISA kits: human TNF-α ELISA kit (ab181421, Abcam, USA), rat TNF-α ELISA kit (ab100785, Abcam), human IL-1β ELISA kit (ab214025, Abcam), rat IL-1β ELISA kit (ab100768, Abcam), human IL-6 ELISA kit (ab46027, Abcam), rat IL-6 ELISA kit (#MBS2021530, MyBioSource), human IL-10 ELISA kit (EK0416, Boster, USA) and rat IL-10 ELISA kit (EK0418, Boster).

### Intestinal microbiota analysis

Stool samples were collected and immediately placed into liquid nitrogen for preservation. Bacterial DNA was extracted from the fecal samples, and the DNA concentration and purity were assessed using a NanoDrop 2000 UV-vis spectrophotometer (Thermo Fisher Scientific, USA). PCR amplification of the bacterial 16S rDNA V3-V4 region was conducted using primers 338 F 5′-ACTCCTACGGGAGGCAGCAG-3′ and 806R 5′-GGACTACHVGGGTWTCTAAT-3′ on a GeneAmp 9700 PCR system (ABI, USA). The Illumina HiSeq2000 sequencing technology was employed to pool and sequence the PCR products following standard protocols. The composition of bacterial communities was analyzed at both the phylum and genus levels.

### Western blot

Total protein was extracted from uterine tissues and HESCs using RIPA lysis buffer and quantified using a BCA protein detection kit (Beyotime, China). Equal amounts of cell protein were separated by 12% SDS-PAGE gel, and then transferred to PVDF transfer membranes. After washing with TBST, the samples were blocked with TBST containing 5% skimmed milk powder for 1 h. Subsequently, the membranes were subjected to incubation with a designated primary antibody at a temperature of 4℃ overnight. The following primary antibodies were used: human/rat Anti-TLR4 antibody (ab22048, 1/1000, Abcam), human Anti-NF-kB p65 antibody (ab16502, 0.5 µg/mL, Abcam), rat Anti-NF-kB p65 antibody (ab19870, 2.5 µg/mL, Abcam), human/rat Anti-NF-kB p65 (phospho S536) (ab76302, 1/1000, Abcam), human/rat Anti-NLRP3 antibody (ab264468, 1/1000, Abcam). After washing, a secondary antibody (ab97196, 1/1000, Abcam) was applied at room temperature for a period of 45 min. Immunoreactive signals were visualized and analyzed using a Tanon-4500 digital image processing system (Tanon, China) and Image-Pro Plus software (Media Cybernetics, Inc., USA).

### qRT-PCR

To extract total RNA from uterine tissues and HESCs, Trizol reagent (Invitrogen, USA) was applied in this procedure. The conversion of mRNA into cDNA was performed using PrimeScript RT Master Mix (Takara Bio, Japan). mRNA expression was quantified in an ABI 7500 System (Applied Biosystems, USA) with TB Green^®^ Premix Ex Taq™ II (Takara, Japan). The primer sequences are listed below:

- TNF-α F 5′-CCTCTCTCTAATCAGCCCTCTG-3′,
-         R 5′-GAGGACCTGGGAGTAGATGAG-3′;
- IL-1β F 5′-ATGATGGCTTATTACAGTGGCAA-3′,
- R 5′-GTCGGAGATTCGTAGCTGGA-3′;
- IL-6 F 5′-ACTCACCTCTTCAGAACGAATTG-3′,
-        R 5′-CCATCTTTGGAAGGTTCAGGTTG-3′;
- IL-10 F 5′-GACTTTAAGGGTTACCTGGGTTG-3′,
- R 5′-TCACATGCGCCTTGATGTCTG-3′;
- GAPDH F 5′-GGAGCGAGATCCCTCCAAAAT-3′,
-             R 5′-GGCTGTTGTCATACTTCTCATGG-3′.

The fold changes of target mRNA expression were calculated using 2^–ΔΔCT^ method, with GAPDH serving as the endogenous control.

### Statistical analysis

All data were presented as mean ± SD and analyzed using GraphPad Prism 7.0. Comparison among multiple groups were analyzed by one-way analysis of variance (ANOVA), followed by Tukey’s post hoc test. *P* < 0.05 was considered to be statistically significant.

## Results

### TGP attenuated the uterine structural pathological changes and inflammation in CE rats

**Fig. 1:**
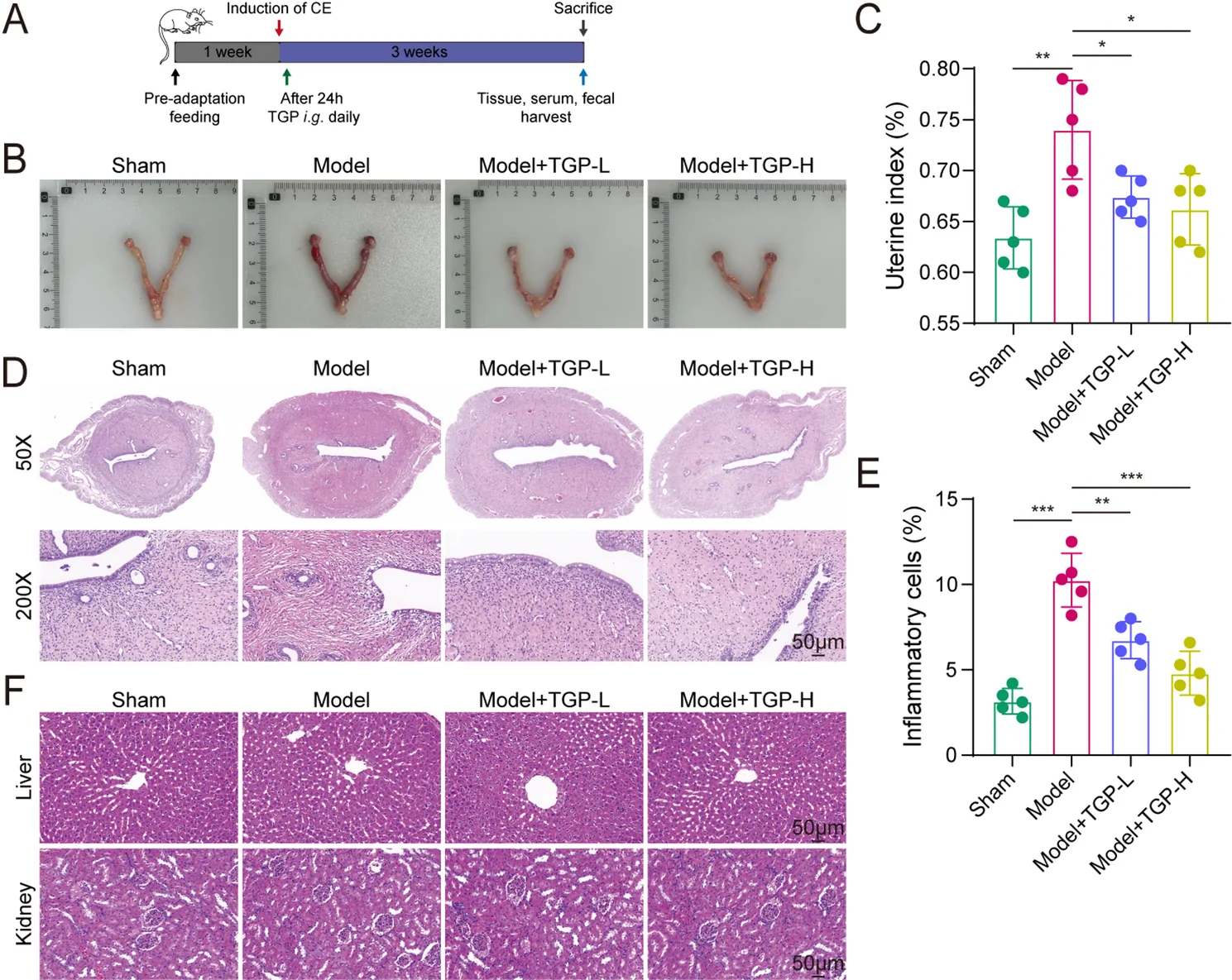
TGP attenuated the uterine structural pathological changes and inflammation in CE rats. (**A**) The rats were divided into four groups: sham; model; model + TGP-L (150 mg/kg); model + TGP-H (300 mg/kg). After one week of pre-adaptation feeding, the rats were intravaginally infused with 60 µL of 5.0 mg/mL LPS to establish the CE model. After 24 h of CE induction, TGP was administered daily to the rats (150 or 300 mg/kg) via a stomach tube for three weeks. The rats were sacrificed and tissues, serum and fecal samples were harvested. (**B-C**) Representative images of rat uterus and quantification of uterine index. (**D-E**) Pathological changes in uterine tissue were detected using H&E staining. The inflammatory cells were quantified. (**F**) Pathological changes in kidney and liver tissues were detected using H&E staining. *P < 0.05, **P < 0.01, ***P < 0.001

To investigate the functional role of TGP in CE, a CE rat model was established, and the rats were treated with doses of 150 mg/kg (low dose) and 300 mg/kg (high dose) of TGP. The experimental schedule is shown in Fig. 1A. As depicted in Fig. 1B-C, no abnormalities in the rat uterus morphology were observed in the sham group, while nodular swelling was observed in both the uterus and vagina of rats in model group, the degree of which was gradually decreased in a TGP concentration-dependent manner. In contrast to the sham group, the model group exhibited damaged uterine tissue, with edema in the endometrium, accompanied by a large number of infiltrated inflammatory cells. Notably, TGP treatment at different concentrations relieved uterine tissue damage in CE rats (Fig. 1D-E). Next, we analyzed the toxic effects of TGP on rats. HE staining suggested that TGP did not induce significant toxic effects and alterations in the morphology of liver and kidney tissues in rats (Fig. 1F). These results demonstrated that TGP exerted a protective role in CE rats.

### TGP alleviated inflammation in CE rats through suppressing the TLR4/NF-κB/NLRP3 signaling pathway

**Fig. 2:**
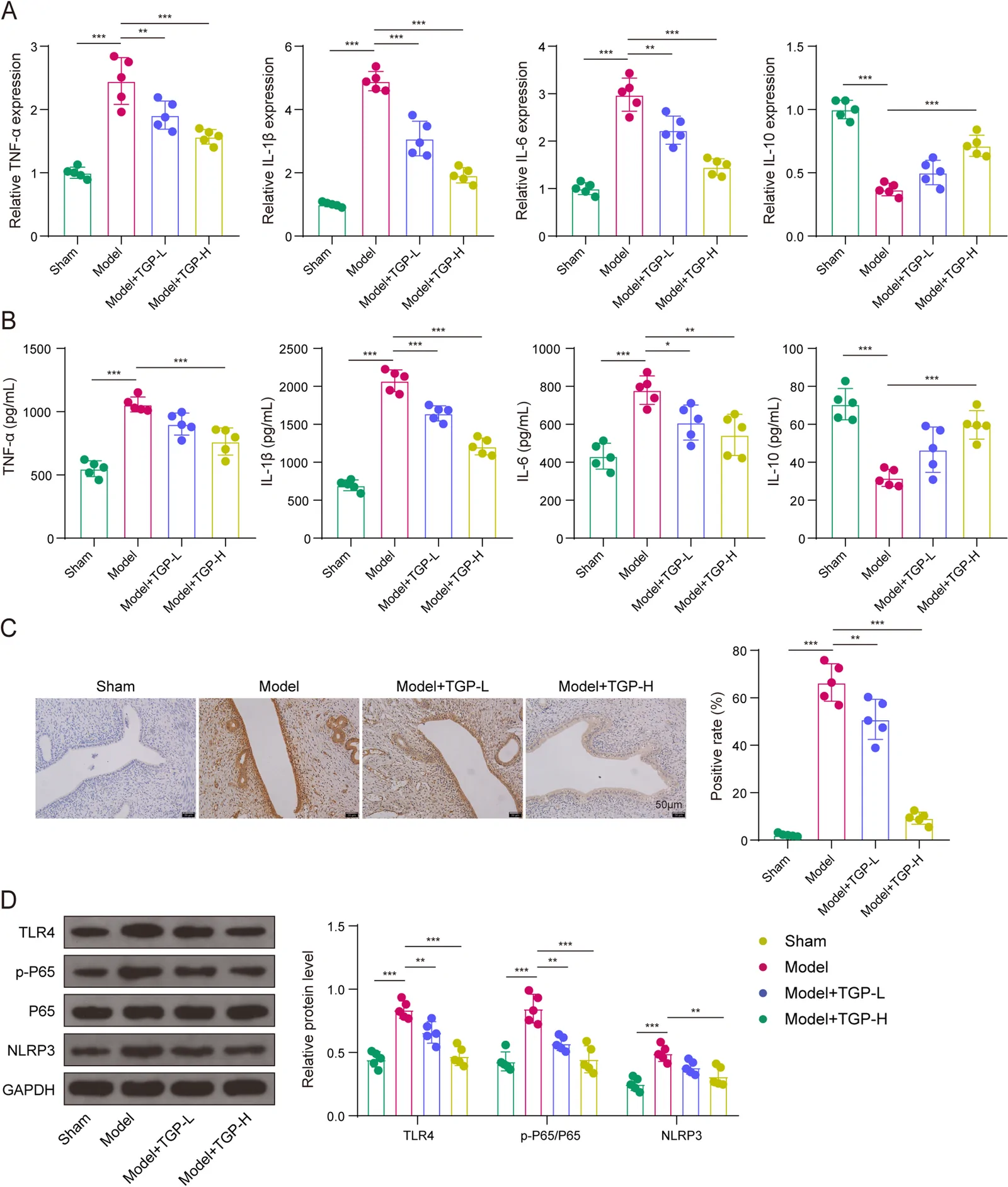
TGP alleviated inflammation in CE rats through inhibiting the TLR4/NF-κB/NLRP3 signaling pathway. The rats were divided into four groups: sham; model; model + TGP-L (150 mg/kg); model + TGP-H (300 mg/kg). (**A**) TNF-α, IL-1β, IL-6 and IL-10 in uterine tissues were detected using qRT-PCR. (**B**) TNF-α, IL-1β, IL-6 and IL-10 in uterine tissues were detected using ELISA assay. (**C**) Protein NLRP3 in uterine tissues was determined using IHC. (**D**) TLR4 and NLRP3, and the phosphorylation of p65 in rats were analyzed using western blot. *P < 0.05, **P < 0.01, ***P < 0.001

Recent evidence shows that the release of inflammatory cytokines is mediated via TLR4/NF-κB/NLRP3 pathway [20]. CE rats were intraperitoneally injected with TLR4 inhibitor TAK-242 (3 mg/kg) to suppress TLR4/NF-κB/NLRP3 signaling. Compared with the model group, the levels of TLR4 and NLRP3 protein and P65 phosphorylation were reduced in uterine tissues of CE rats treated with TAK-242 (Figure S1A). Additionally, TAK-242 injection repressed the release of pro-inflammatory factors TNF-α, IL-1β and IL-6 but promoted IL-10 production in CE rats (Figure S1B). These findings suggested that the suppression of TLR4/NF-κB/NLRP3 signaling could attenuate inflammation in CE. Next, the modulation of TGP intervention on the inflammatory response was analyzed in CE rats. The data presented in Fig. 2A-B illustrated that TGP intervention at different doses suppressed the secretion of pro-inflammatory factors, suggesting the anti-inflammatory effects of TGP in CE rats. To further elucidate the potential molecular mechanisms of TGP in CE rats, we assessed the activity of the TLR4/NF-κB/NLRP3 pathway. The IHC results indicated that the expression of NLRP3 protein was significantly increased in model group compared with the sham group, while both the low and high dose of TGP inhibited the expression of NLRP3 protein in CE rats (Fig. 2C). Additionally, the western blot analysis further revealed inhibitory effects of TGP on the expression of TLR4 and NLRP3, as well as the phosphorylation of p65 in CE rats (Fig. 2D), suggesting a suppressive impact of TGP on the TLR4/NF-κB/NLRP3 pathway. These findings demonstrated that TGP alleviated the inflammatory response in CE rats through inhibiting the TLR4/NF-κB/NLRP3 signaling pathway.

### Regulation of TGP for the intestinal microbiota of CE rats

**Fig. 3:**
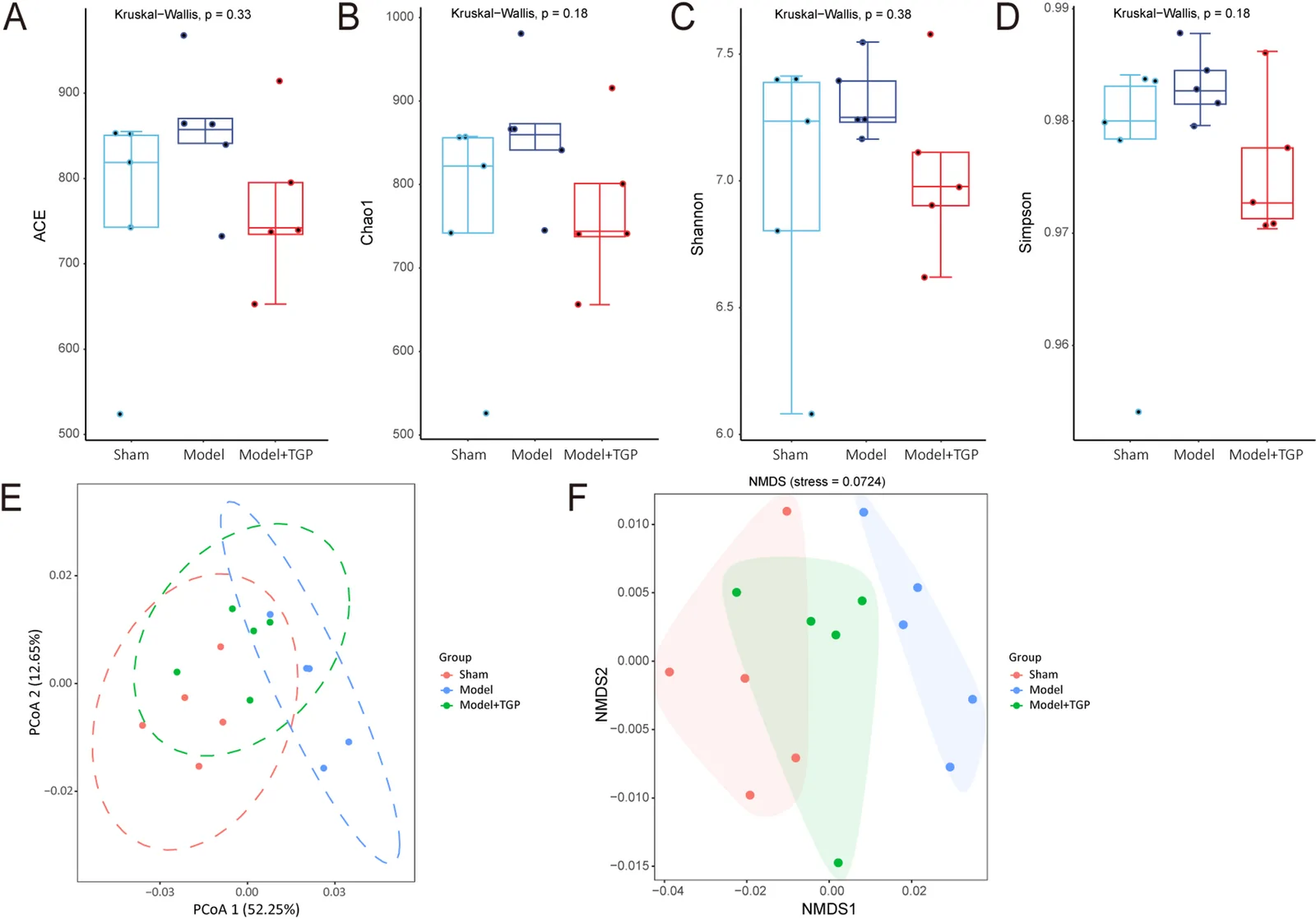
Regulation of TGP for the intestinal microbiota of CE rats. The rats were divided into three groups: sham; model; model + TGP-H (300 mg/kg). (**A-D**) The ACE indexes (**A**), Chao1 indexes (**B**), Shannon indexes (**C**) and Simpson indexes (**D**). (**E-F**) The variance in microbial composition is analyzed using PCoA (**E**) and NMDS (**F**)

To elucidate the potential beneficial effects of TGP on CE rats via the intestinal microbiota, 16S rRNA sequencing was conducted in this study. Alpha-diversity indicators, including Chao1 index, Shannon index, ACE index and Simpson index, exhibited similar trends across the different indices in the Sham, CE and TGP-treated CE groups (Fig. 3A-D). Moreover, based on weighted UniFrac, PCoA and NMDS analyses revealed that although there was overlapping among Sham, CE, and TGP treatment groups, there were differences in the microbial compositions among these three groups (Fig. 3E-F). These data suggested that TGP intervention altered the composition of gut microbiota in CE rats.

### TGP induced changes in the specific bacterial taxa in CE rats

**Fig. 4:**
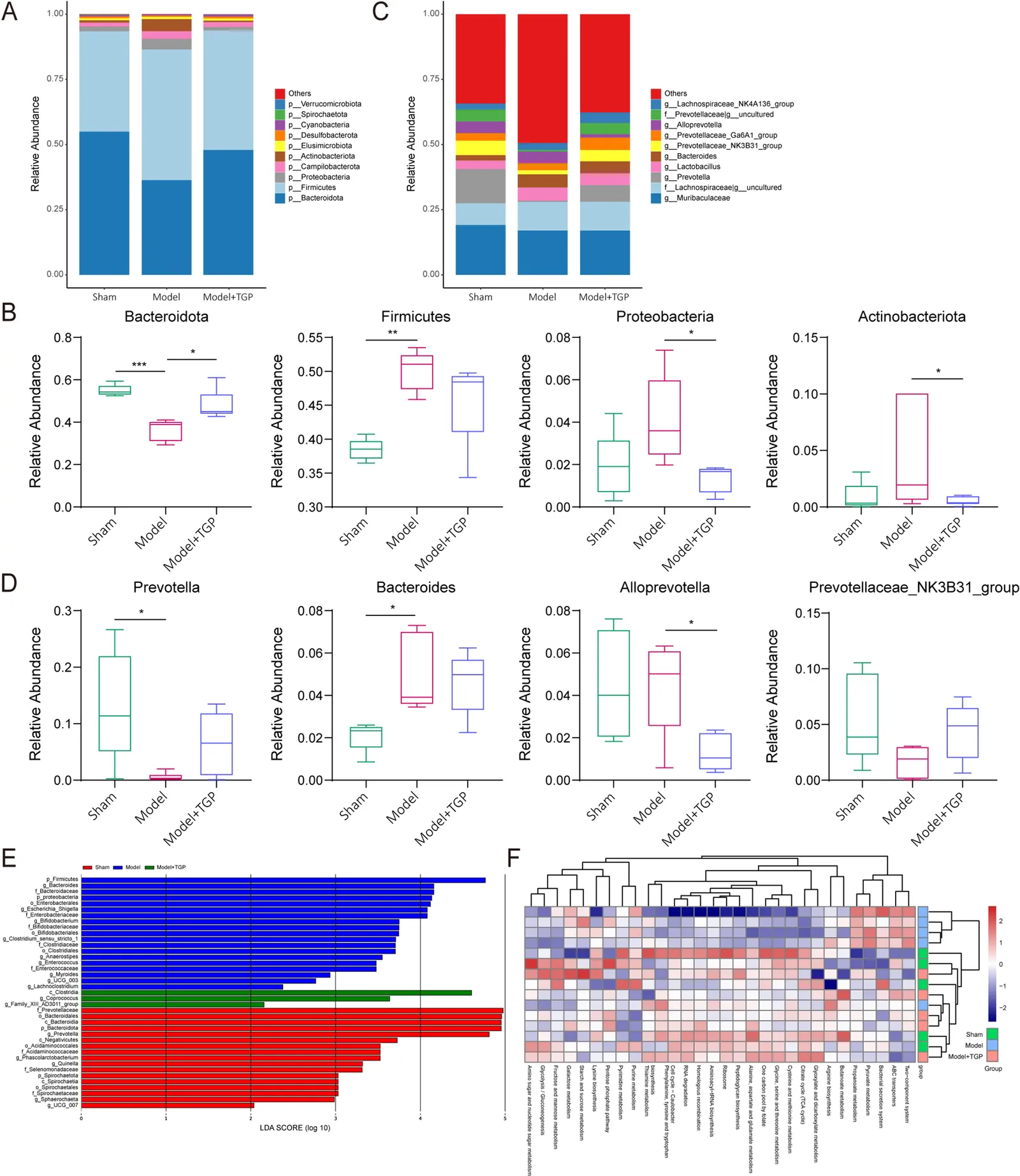
TGP induced changes in the specific bacterial taxa in CE rats. (**A-B**) Quantitative data of relative abundance at the phylum level of gut microbiota in sham group, model group, model + TGP-H (300 mg/kg) group. (**C-D**) Quantitative data of relative abundance at the genus level of gut microbiota. (**E**) Differentially enriched intestinal microbiota across all groups were identified using linear discriminant analysis (LDA). (**F**) Heatmap showed differential metabolic pathways among three groups. *P < 0.05, **P < 0.01, ***P < 0.001

To further confirm the regulating effect of TGP on gut microbiota diversity in CE rats, the taxonomic profile of gut bacteria was analyzed at both the phylum and genus levels. At the phylum level (Fig. 4A-B**)**, the abundance of Bacteroidota was reduced and the abundance of Firmicutes was increased in the model group compared with the sham group, while TGP treatment increased the abundance of Bacteroidota and reduced the abundance of Proteobacteria and Actinobacterota in CE rats. At the genus level (Fig. 4C-D), the CE group exhibited a notable decrease in the abundance of Prevotella and increase in the abundance of Bacteroides, while TGP intervention down-regulated the abundance of Alloprevotella in CE rats. Additionally, the intestinal microbiota in three groups was analyzed using LDA. As depicted in Fig. 4E, a total of 39 species exhibited significant differences among the three groups. Notably, Firmicutes, Clostridia, and Prevotellaceae were identified as key microbial markers for the sham group, model group, and model + TGP group, respectively, highlighting their differential abundance. Additionally, a heatmap indicated differential metabolic pathways among three groups (Fig. 4F). These findings indicated that TGP modulated the composition of the intestinal microbiota of CE rats.

### TGP inhibited inflammation in LPS-induced HESCs

**Fig. 5:**
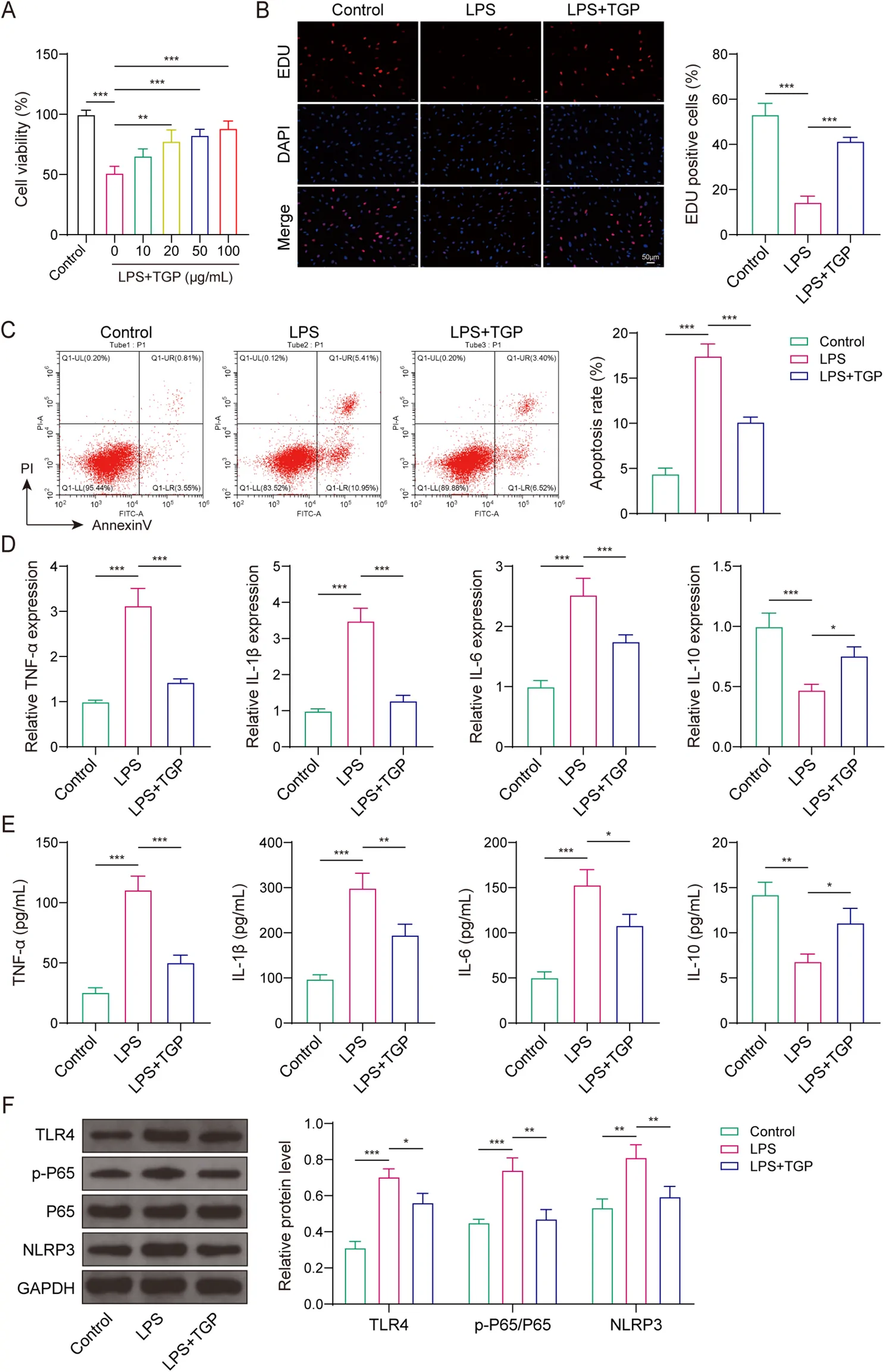
TGP inhibited inflammation in LPS-induced HESCs. (**A**) LPS-stimulated HESCs were treated with TGP at different concentrations of 0, 10, 20, 50 and 100 µg/mL. cell viability was evaluated using CCK-8 kit. (**B**) Cell proliferation was assessed by EDU staining. (**C**) Cellular apoptosis was evaluated using flow cytometry. (**D-E**) Inflammatory cytokines TNF-α, IL-1β, IL-6 and IL-10 in HESCs were quantified using qRT-PCR and ELISA. (**F**) TLR4 and NLRP3, and the phosphorylation of p65 in HESCs were analyzed using western blot. *P < 0.05, **P < 0.01, ***P < 0.001

Finally, we investigated the regulatory effect of TGP on LPS-stimulated HESCs. LPS-stimulated HESCs were exposed to varying concentrations of TGP (0, 10, 20, 50 and 100 µg/ml). Our results demonstrated that LPS treatment significantly suppressed cell viability, which was dose-dependently reversed by TGP exposure (Fig. 5A). Additionally, EDU staining revealed that TGP treatment rescued LPS-induced inhibition of the proliferation rate of HESCs (Fig. 5B). Consistently, TGP reduced cellular apoptosis in LPS-induced HESCs (Fig. 5C). Moreover, both qRT-PCR and ELISA assays showed that TGP intervention significantly reduced the release of pro-inflammatory cytokines induced by LPS (Fig. 5D-E). Furthermore, TGP treatment led to notable suppression of the proteins of TLR4, NF-κB and NLRP3, illustrating its inhibitory impact of TGP on inflammation in the CE cell model (Fig. 5F). In summary, TGP treatment effectively inhibited inflammation in LPS-induced HESCs.

## Discussion

Currently, CE is commonly managed with antibiotics to eradicate the underlying infection, and severe cases may necessitate surgical intervention [21]. However, the current therapeutic approaches have shown limited efficacy and safety. The dysbiosis of gut microbiota may contribute to immune system dysregulation, potentially increasing the risk of developing CE [22]. Numerous studies have demonstrated the anti-inflammatory properties of TGP in the context of chronic inflammation. This investigation revealed that TGP mitigated chronic endometritis in rats by regulating intestinal microbiota composition and inhibiting the TLR4/NF-κB/NLRP3 pathway.

The suppressing effects of TGP on inflammation have been reported across various diseases. Paeoniflorin, the primary active component of TGP, has exhibited anti-inflammatory properties in animal models of autoimmune diseases by modulating immune cell function, reducing the production of inflammatory mediators, restoring aberrant signaling pathways, and maintaining balance among immune cell subsets [23]. A previous study demonstrated the effective improvement of allergic rhinitis symptoms, histopathological changes, oxidative stress, cell apoptosis, and inflammatory response through Smad7-TGF-β pathway upon TGP treatment [24]. These findings collectively suggest that TGP may possess potential anti-inflammatory effects on endometritis. Consistent with this, our investigation proved that TGP suppressed the release of TNF-α, IL-1β and IL-6 in CE, indicating its suppressive effect on inflammation. Emerging evidence elucidates that the dysregulated activation of the TLR4/NF-κB/NLRP3 signaling pathway in endometritis results in sustained inflammation and tissue damage within the endometrium. Reportedly, Oxycodone exposure restrained the production of inflammatory cytokines in mifepristone-induced human endometrial stromal cells via the TLR4/NF-κB Pathway, resulting in the inhibition of cell death and endometrial injury [25]. Furthermore, another study revealed that the inhibition of the TLR4-NLRP3 signaling pathway exerted a protective effect on endometritis [26]. Notably, our study confirmed the activation of the TLR4/NF-κB/NLRP3 pathway in CE. Moreover, it was reported that paeoniflorin induced significant suppression of the TLR4/NF-κB/NLRP3 axis and pro-inflammatory mediators in the hippocampus of LPS-stimulated mice [27]. In line with these findings, our study identified that TGP administration inhibited the TLR4/NF-κB/NLRP3 pathway in the CE model.

The intestinal mucosal barrier, a critical defense against pathogens, relies on the symbiotic balance of gut microbiota [28]. The dysbiosis of gut microbiota, characterized by the loss of beneficial microbes and overgrowth of pathogenic microbiota, compromises the colonic mucus layer, enabling pathogenic infiltration and systemic dissemination of bacteria and endotoxins [29]. The disruption of gut microbiota composition increases the risk of pathogen infection, triggers the overgrowth of harmful pathobionts, and contributes to the progression of inflammatory diseases [30], including CE. For instance, Xue et al. revealed that alterations in gut microbiota influenced metabolic pathways in endometritis, thereby exerting regulatory effects on inflammatory response [31]. Hu et al.’s study indicated that gut microbiota attenuated endometritis by regulating the levels of short-chain fatty acids and maintaining neutrophil functionality [32]. The modulation of intestinal microflora improved the uterine structural pathological changes and decreased the generation of pro-inflammatory cytokines in LPS-induced endometritis in mice [33]. In our study, CE rats exhibited severe uterine structural damage alongside altered gut microbial composition and elevated pathogenic abundance. These findings align with the emerging concept of gut-uterus axis mechanism, where gut-derived inflammatory signals exacerbate uterine pathology [34].

Notably, TGP intervention reversed these pathological trends. TGP-treated CE rats displayed alleviated uterine tissue damage and decreased pro-inflammatory cytokines (TNF-α, IL-1β and IL-6). These findings were supported by previous studies. The study of Yan et al. illustrated that PRA effectively alleviated colitis in mice by modulating the integrity of gut barrier and microbiota, and immune reactions [35]. Another study indicated that TGP treatment augmented the relative abundance of specific beneficial symbiotic bacteria, thereby altering taxonomic disparities in microbial composition in arthritis rats [36]. Similarly, this study revealed that TGP intervention modulated the composition of gut microbiota in CE rats. Additionally, TGP was found to promote the proliferation of essential beneficial bacteria while suppressing the growth of predominant pathogenic bacteria, thereby enhancing the richness and distribution of gut microorganisms in mice with Sjögren’s syndrome [37]. Conformably, our results verified that TGP treatment reduced the abundance of Proteobacteria and Actinobacterota in CE rats, indicating TGP may exert its protective effect by affecting the viability of beneficial or pathogenic bacteria in the gut. Li et al. found that the abundance of Alstipes, Bacteroides, Phascolarctobacterium, Romboutsia and Prevotella was down-regulated in a bovine endometritis model [38]. Another study revealed that alterations in Sphingomonas, Alloprevotella, and Acinetobacter contributed to the changes in community structure in endometrial microbiota [39]. Our study revealed that TGP intervention decreased the abundance of Alloprevotella at genus level. These findings suggest that TGP may exert its protective effects on gut health through modulation of gut microbiota composition and function.

Taken together, this study elucidated the functions and the regulatory mechanisms of TGP in CE. Our study corroborated that TGP intervention alleviated chronic endometritis in rats by modulating gut microbiota composition and inhibiting of the TLR4/NF-κB/NLRP3 pathway. These results imply that TGP could serve as a potential agent for the treatment of CE.

## Supplementary Information

Below is the link to the electronic supplementary material.

Supplementary Material 1

## Data Availability

All data generated or analyzed during this study are included in this published article.
